# Brain development in newborns and infants after ECMO

**DOI:** 10.1007/s12519-023-00768-w

**Published:** 2024-01-19

**Authors:** Kai Yan, Lu-Kun Tang, Fei-Fan Xiao, Peng Zhang, Guo-Qiang Cheng, Lai-Shuan Wang, Chun-Mei Lu, Meng-Meng Ge, Li-Yuan Hu, Yuan-Feng Zhou, Tian-Tian Xiao, Yan Xu, Zhao-Qing Yin, Gang-Feng Yan, Guo-Ping Lu, Qi Li, Wen-Hao Zhou

**Affiliations:** 1 Department of Neonatology, Children Hospital of Fudan University, Shanghai, 201102 China; 2 Kunming Medical University Affiliated Dehong Hospital, Dehong, Yunnan China; 3 Graduate School, Kunming Medical University, Kunming, Yunnan China; 4 Department of Neurology, Children’s Hospital of Fudan University, Shanghai, China; 5 School of Medicine, Chengdu Women’s and Children’s Central Hospital, University of Electronic Science and Technology of China, Chengdu, Sichuan China; 6 Department of Intensive Care Medicine, Children’s Hospital of Fudan University, Shanghai, China; 7 Department of Intensive Care Medicine, The Sixth Medical Center of PLA General Hospital, Beijing, China; 8 Key Laboratory of Neonatology, National Health Care Commission, Shanghai, China

**Keywords:** Brain development, Extracorporeal membrane oxygenation, Infants, Neonates, Neurological outcomes

## Abstract

**Background:**

Extracorporeal membrane oxygenation (ECMO) not only significantly improves survival rates in severely ill neonates but also is associated with long-term neurodevelopmental issues. To systematically review the available literature on the neurodevelopmental outcomes of neonates and infants who have undergone ECMO treatment, with a focus on motor deficits, cognitive impairments, sensory impairments, and developmental delays. This review aims to understand the incidence, prevalence, and risk factors for these problems and to explore current nursing care and management strategies.

**Data sources:**

A comprehensive literature search was performed across PubMed, EMBASE, and Web of Science using a wide array of keywords and phrases pertaining to ECMO, neonates, infants, and various facets of neurodevelopment. The initial screening involved reviewing titles and abstracts to exclude irrelevant articles, followed by a full-text assessment of potentially relevant literature. The quality of each study was evaluated based on its research methodology and statistical analysis. Moreover, citation searches were conducted to identify potentially overlooked studies. Although the focus was primarily on neonatal ECMO, studies involving children and adults were also included due to the limited availability of neonate-specific literature.

**Results:**

About 50% of neonates post-ECMO treatment exhibit varying degrees of brain injury, particularly in the frontal and temporoparietal white matter regions, often accompanied by neurological complications. Seizures occur in 18%–23% of neonates within the first 24 hours, and bleeding events occur in 27%–60% of ECMO procedures, with up to 33% potentially experiencing ischemic strokes. Although some studies suggest that ECMO may negatively impact hearing and visual development, other studies have found no significant differences; hence, the influence of ECMO remains unclear. In terms of cognitive, language, and intellectual development, ECMO treatment may be associated with potential developmental delays, including lower composite scores in cognitive and motor functions, as well as potential language and learning difficulties. These studies emphasize the importance of early detection and intervention of potential developmental issues in ECMO survivors, possibly necessitating the implementation of a multidisciplinary follow-up plan that includes regular neuromotor and psychological evaluations. Overall, further multicenter, large-sample, long-term follow-up studies are needed to determine the impact of ECMO on these developmental aspects.

**Conclusions:**

The impact of ECMO on an infant’s nervous system still requires further investigation with larger sample sizes for validation. Fine-tuned management, comprehensive nursing care, appropriate patient selection, proactive monitoring, nutritional support, and early rehabilitation may potentially contribute to improving the long-term outcomes for these infants.

## Introduction

**Fig. 1:**
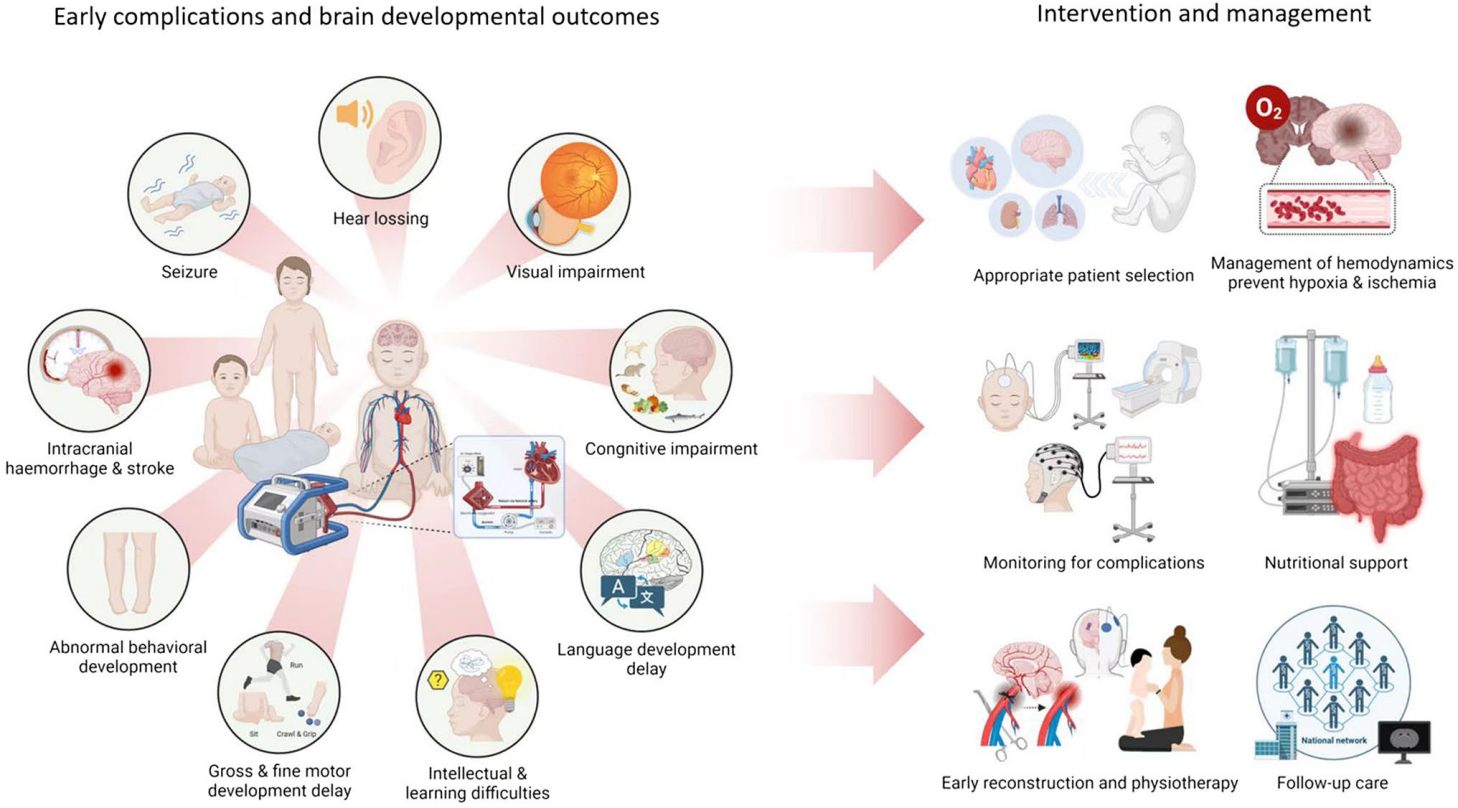
Early complications, brain developmental outcomes, intervention and management

Extracorporeal membrane oxygenation (ECMO) is a life-preserving method employed for critically ill neonates needing respiratory or cardiac support [1, 2]. This technique utilizes a pump and an oxygenator to bypass the heart and lungs of the neonate, providing a temporary support system that permits these vital organs to recover. While ECMO has been instrumental in boosting survival rates in these severely ill neonates, it is also linked to an array of long-term neurodevelopmental issues [3–8]. These issues span from motor and cognitive impairments to sensory issues and developmental delays. In this review, we aim to explore the neurodevelopmental issues associated with ECMO. Our focus is on the incidence of these complications among neonates undergoing ECMO and the potential risk factors contributing to their development. In addition, we examine the current nursing and clinical management strategies used for neonates post-ECMO treatment, aiming to minimize the risk of neurodevelopmental complications (Fig. 1). Our goal is to offer a comprehensive understanding of the neurodevelopmental challenges that may be faced by neonates following ECMO treatment, as well as potential solutions to these challenges.

### Literature search and review

We conducted literature searches through the PubMed, EMBASE, and Web of Science databases for this study. The search strategy involved keywords and phrases such as “Extracorporeal Membrane Oxygenation,” “ECMO,” “Newborns,” “Neonates,” “Neurodevelopment,” “Hearing,” “Vision,” “Cognitive,” “Seizures,” “Stroke,” “Motor development,” “Language development,” “Neuromotor outcomes,” “Neurocognitive outcomes,” and “Neurosensory outcomes.” These terms were combined using “AND” and “OR” Boolean operators, focusing on aspects related to brain development associated with ECMO in neonates. Following the initial search results, we screened the titles and abstracts of the articles, excluding those that were not related to ECMO. All potentially relevant literature was fully assessed to ascertain whether it was aligned with our research topic and criteria. We did not exclude studies involving children due to the limited number of articles relating to neonatal–infant ECMO. We also retained some adult ECMO studies with larger sample sizes. Each study was subjected to a quality assessment, mainly focusing on research methods and the appropriateness of statistical analysis. To ensure the accuracy of the literature search, we manually inspected the citations of all selected articles to identify potentially overlooked related studies.

### Neurological complications

Neurological complications represent the most common form of neurological impairment encountered during the ECMO process. Despite the rise in ECMO usage over the past decade, the incidence of reported neurological complications remains unchanged. A study on neonates treated with ECMO over a 10-year period found that magnetic resonance imaging (MRI) revealed brain injuries in nearly half of these neonates after their treatment [9]. The frontal and temporoparietal white matter were the most commonly affected areas. Furthermore, the frequency of brain injuries appeared to be higher in neonates who received the venoarterial mode of extracorporeal membrane oxygenation [9]. The complications include seizures, ischemic strokes, and intracranial hemorrhages.

Seizures have been identified in 18%–23% of neonates and children undergoing ECMO treatment [10–12]. Among all monitored patients, the incidence of electrographic seizures without clinical symptoms ranges from 12% to 16% [10–12]. Most of these seizures occurred within the first 24 hours of ECMO monitoring and were associated with higher mortality rates and unfavorable neurodevelopmental outcomes [13]. These seizures often correlated with intracranial pathology visible on head imaging. The July 2019 international report from the Extracorporeal Life Support Organization (ELSO) registry showed electroencephalogram (EEG)-confirmed seizure rates ranging from 1.9% in pediatric respiratory ECMO patients to 11.4% in neonates who underwent emergency cardiopulmonary resuscitation (ECPR) [14]. The ELSO is dedicated to improving patient survival after ECMO treatment. The neonatal ELSO registry, a component of the overall ELSO registry [15], has established a global ECMO database. Currently, the number of children and neonates registered in ELSO for ECMO reaches 85,595 and 48,771, respectively. This organization collects data on neonates receiving ECMO treatment, including details about the treatment type, duration, complications, and outcomes, that can provide critical information to help physicians and researchers understand the complications of ECMO treatment in neonates and improve treatment strategies [16].

However, these rates are likely underestimated, as suggested by single-center studies with active EEG monitoring. Two recently published single-center studies reported substantially higher rates of seizures under EEG monitoring in pediatric patients on ECMO, exceeding the rates reported by ELSO [17, 18]. One study focusing on the neonatal population reported a seizure rate of 18% (18/99) in neonates on ECMO under EEG monitoring [11]. Another single-center study reported a particularly high seizure rate of 40% (18/45) in infants under one year old on ECMO [19]. The causes of seizures during ECMO are multifaceted and could be attributed to hypoxic–ischemic injury from cardiac arrest, intracranial hemorrhage, or ischemic stroke [20]. Seizures increase the risk of unfavorable short-term neurological outcomes, death, and long-term complications such as cerebral palsy and cognitive deficits [13, 20, 21]. Given the frequency and potential impact of seizures, it is imperative to monitor patients on ECMO with EEG to promptly detect and treat any seizures.

Despite advancements in hemostasis and anticoagulation management, bleeding events occur in up to 27%–60% of ECMO runs [14, 22, 23], including intracranial hemorrhage, surgical site bleeding, gastrointestinal and pulmonary hemorrhage, and cannulation site bleeding. An early study reported the incidence rate of intracerebral hemorrhage (ICH) in ECMO patients, which included 54 pediatric cases, and the result was 37% (45/123) [24]. A systematic review focusing on the incidence and prognosis of ECMO-associated ICH was conducted. A systematic review incorporated 25 pertinent studies, the findings of which showed considerable variation in the incidence of ECMO-related ICH, ranging from 1.8% to 21% [25], with corresponding mortality rates in the afflicted cohorts varying between 32% and 100%. More recent findings have indicated a slight decrease in the incidence of ICH in neonatal ECMO compared to data from 20 years ago (from 36% down to 28%) [26, 27]. However, potential bias in the data may stem from heterogeneity within the study populations. Given that the occurrence of intracranial hemorrhage during ECMO is closely associated with high mortality, it is critically important to establish a standardized and systematic ICH screening process for these pediatric patients. MRI may also detect cerebral microbleeds post-ECMO [28, 29], with varying clinical significance and phenotypes [30, 31]. ICH appears to be most common within the first four–five days of ECMO. This complication has been linked to increased mortality and worse functional neurological outcomes [22, 32]. The risk factors for ICH on ECMO include pre-ECMO cardiac arrest, sepsis, use of renal replacement therapy, pre-ECMO duration of mechanical ventilation and sudden changes in PaO_2_ and PaCO_2_ with the initiation of VV ECMO [28]. Obstruction of venous outflow from cannulation of the right internal jugular vein may also increase the risk of ICH due to cerebral venous congestion [33]. Coagulation profiles were not found to be predictive of acute ICH or infarct on ECMO in a single-center study [34].

Ischemic strokes can present a significant issue for pediatric patients undergoing ECMO, with up to 33% of these patients potentially having experienced this condition [35–37]. However, the incidence of ischemic strokes reported in the ELSO registry is much lower, ranging from 2% to 7.2% [14]. The standard medical care for ECMO, including the use of sedation and the potential need for neuromuscular blockade, as well as postcannulation patient positioning, can make it difficult to detect early signs of a stroke. Risk factors for stroke during ECMO include circuit thrombosis with the dislodgement of emboli and alterations in cerebral arterial circulation with carotid artery cannulation. However, the evidence on whether carotid cannulation increases the risk of ischemic stroke is mixed [38, 39]. Ischemic strokes can have serious consequences for pediatric patients on ECMO, and it is important to have measures in place to detect and prevent these events. In identifying ischemic strokes during ECMO, quantitative electroencephalography (qEEG) monitoring may serve as a useful tool. While no direct research has indicated that qEEG can prevent ischemic strokes during ECMO, some studies have suggested its potential value in detecting the early onset of ischemic strokes. It has also been valuable for monitoring neurological issues such as seizures in critically ill patients, which could indirectly contribute to the timely identification and management of issues that may lead to stroke [40, 41]. Additionally, maintaining optimal cardiovascular function [42], careful patient selection and device strategy [43], careful cannulation technique and monitoring [44], and novel antithrombotic strategies [45] may all play a role in preventing ischemic strokes during ECMO. The combined application of these strategies and techniques may help to minimize the risk of ischemic stroke in ECMO patients.

### Hearing development

ECMO is a complex procedure, and the full extent of its effects on neonatal hearing development remains unclear. Several studies have investigated the effects of ECMO on hearing development, and the results have been conflicting. Some studies have found that ECMO has a negative effect on hearing development [46, 47], while other studies have found that ECMO cannot yet be considered to be associated with hearing loss [48].

In a study conducted by the Children’s Hospital of Michigan, the authors investigated the prevalence of hearing loss in 15 neonates who underwent ECMO and were followed up for hearing assessment. The results showed that 53% (8/15) of the neonates had hearing loss, which is significantly higher than the prevalence of hearing loss in the general population. The authors concluded that ECMO can be a risk factor for hearing loss in neonates [49]. However, this is a small sample study with only 15 participants, and its conclusions still need to be confirmed by homogeneous research with a larger sample size. Similarly, a study conducted by the Children’s Hospital of Boston investigated the effects of ECMO on hearing development in neonates. Twenty patients were treated with ECMO. Neurological problems were common in these patients: seven children (21%) required hearing aids, and seven others had abnormal results with brainstem auditory evoked response (BAER) testing [50].

A retrospective study aimed to determine the incidence of sensorineural hearing loss (SNHL) in neonatal ECMO survivors between the ages of 9 and 13 years and to investigate the association between neonatal ECMO and SNHL [51]. The medical records of 212 neonatal ECMO survivors were analyzed to identify children with complete hearing evaluation at 9–13 years of age. Of the 48 patients who had a complete hearing evaluation, eight were diagnosed with SNHL, and two patients developed SNHL. The duration of ECMO therapy was independently associated with SNHL. These findings indicate that longitudinal neurodevelopmental follow-up is essential for ECMO survivors.

Partridge et al. aimed to investigate the incidence and risk factors associated with SNHL and conductive hearing loss (CHL) in a large cohort of patients with congenital diaphragmatic hernia (CDH) who underwent standardized treatment and follow-up at a single institution. The authors retrospectively reviewed the charts of 112 CDH patients in their pulmonary hypoplasia program from January 2004 through December 2012. They found that 2.7% of patients had SNHL and 34.0% had CHL. The authors identified several risk factors associated with SNHL, including the requirement for ECMO. SNHL occurred in three of the diaphragmatic hernia neonates, and all three of these cases received ECMO (*P* = 0.013). However, no statistically significant association was found between ECMO and the occurrence of conductive hearing loss in neonates with diaphragmatic hernia (*P* = 0.862).

In contrast, in the long-term follow-up of the brain bilaterally, Lott’s study [52] focused on 10 children aged 4–9 years who underwent neonatal ECMO treatment. There was no significant difference observed in the auditory threshold, auditory evoked potential latency, V wave amplitude of automatic auditory brainstem response, auditory P30 wave of evoked potential, or N12 wave amplitude of somatosensory evoked potential. Mahomva et al. investigated the incidence of auditory neuropathy spectrum disorder (ANSD) and its risk factors among the neonatal intensive care unit (NICU) population from 2009 to 2018 [48]. A retrospective national database review was conducted using the Pediatric Health Information System database. The research indicates that ECMO is not a high-risk factor for the occurrence of ANSD in NICU patients. The study included 1128 neonates who underwent ECMO in the NICU, and the results showed that only 0.1% (1/1, 128) of the neonates developed a hearing spectrum disorder. The authors concluded that ECMO does not have a significant effect on hearing development in neonates. Nevertheless, the research team highlighted the need for continued monitoring and management of these high-risk children.

Wilson et al. evaluated the long-term audiological outcomes of CDH survivors at a single tertiary care center with ECMO capability and a neonatal follow-up program. Records of CDH survivors from 2000 to 2010 were analyzed. Audiological surveillance identified only one patient with SNHL (who received high frequency oscillatory, inhaled nitric oxide, and patch repair), suggesting that the incidence of SNHL in this population may be less than previously reported. They considered that the lower incidence might be related to early hearing screening of newborns and that comprehensive testing must be performed until school age to monitor for any long-term hearing loss [53].

Considering that ECMO may be one of the risk factors for hearing loss in newborns, it is advisable that neonates who undergo ECMO treatment should receive thorough hearing screening and follow-up as early as possible to ensure their normal hearing development [54]. The authors have recommended ECMO as a high-risk factor that requires active continuous hearing follow-up, with an initial hearing assessment suggested to be carried out after 3 months [54]. Given that these are small sample studies, there is no definitive conclusion on the details of screening time and follow-up periods. Future research should investigate the long-term effects of ECMO on neonatal hearing development. This will enable healthcare professionals to provide appropriate interventions to prevent or manage hearing impairment in patients as early as possible. Additionally, future research could explore the impact of details such as ECMO duration on a patient’s hearing development from the perspective of adjusting ECMO management strategies.

### Visual development

A retrospective study aimed to evaluate the prevalence of retinal pathologies in neonates with CDH receiving ECMO therapy [55]. The study included 27 infants who received funduscopic examination out of 54 infants treated with ECMO for CDH between 2012 and 2018. Retinal changes were observed in three neonates (11.1%), including multiple midperipheral blot intraretinal hemorrhages in five eyes of three children and retinopathy of prematurity (ROP) in two eyes of one child. All retinal changes regressed without therapeutic intervention. The study concludes that neonates treated with ECMO due to CDH may exhibit retinal hemorrhages, but usually without the need for intervention. Prematurely born infants receiving ECMO may develop ROP and, thus, require ROP screening examinations. Montalva et al., through a meta-analysis, found that children with CDH who received ECMO treatment had a higher incidence of neurodevelopmental impairment, including visual impairment (8%), than those who did not receive ECMO [56].

Another study examined the eyes of 91 neonates treated with ECMO for various conditions [57], including meconium aspiration syndrome, primary persistent pulmonary hypertension, sepsis, CDH, respiratory distress syndrome, and blood aspiration. Asymmetric retinopathy was discovered in six infants with CDH and one infant with respiratory distress syndrome after venoarterial bypass, demonstrating venous tortuosity with or without intraretinal hemorrhages. Patients with CDH had a higher incidence of retinal changes than patients with respiratory distress syndrome or meconium aspiration syndrome. These findings further demonstrated that ECMO-associated retinal vasculopathy disproportionately affects CDH patients who undergo venoarterial bypass and warrant further study to assess its long-term effects.

However, there are some studies with conflicting results. The prevalence of vision-threatening retinal hemorrhages in infants following venoarterial ECMO and any potential correlations with cerebral hemorrhages, thrombocytopenia, carotid re-anastomosis, or death after ECMO were evaluated through a review of dilated ophthalmoscopic examinations performed on 37 infants an average of 16 days after ECMO treatment [58]. Of the 37 infants examined, five (13%) had small intraretinal hemorrhages. The presence or absence of retinal hemorrhages was not linked to cerebral hemorrhage, platelet count, carotid re-anastomosis, or subsequent death. The study concludes that retinal hemorrhages found after ECMO treatment are not necessarily caused by ECMO and may be benign and related to childbirth. Infants undergoing venoarterial ECMO are at a low risk of developing vision-threatening retinal hemorrhages.

The medical records of 171 infants who received ECMO and underwent routine ophthalmic examination were reviewed [59]. Fundus examination was normal in 302 eyes (88%), and the abnormal retinovascular findings that were present, such as venous dilation or intraretinal hemorrhages, were not considered vision-threatening and required no treatment. No clinically significant retinal findings were identified in the patients who underwent post-ECMO screening. The authors suggest that routine dilated fundus examination may not be cost-effective and may place additional and potentially unnecessary stress on these infants. A retrospective review of ocular examinations of 86 infants who underwent ECMO therapy at Children’s Hospital in Los Angeles between 1987 and 1991 found normal findings in 73 infants [60]. One infant had bilateral retinal vascular tortuosity, and 12 had incidental ocular findings, but there was no evidence of left-sided retinal hemorrhage or tortuosity. The results suggest that left-sided retinal vascular changes after ECMO do not occur or occur only rarely and result in no permanent retinal damage.

Retinal hemorrhages and exudates, venous tortuosity, and asymmetric vascular development have all been reported in relevant studies. However, inconsistent research results currently exist regarding the impact of ECMO on neonatal visual development, and further prospective research is needed to verify this potential hypothesis. This includes comparing ophthalmic examinations before, during, and after ECMO, as well as comparisons among different ECMO etiologies, to more accurately assess the long-term impact of ECMO on visual impairment and explore strategies for prevention. Some specific populations receiving ECMO treatment, such as continued oxygen supplementation or extremely premature infants, who are already at high risk for retinopathy of prematurity (ROP), may need eye examinations to prevent visual complications. Larsen et al. reviewed the timing of the initial ROP screening in neonates on ECMO. The first screening time varied across five studies, ranging from 1.2 to 45.8 days after initiation of ECMO treatment [55]. There is no consensus on the timing of the first ROP screening in the neonatal ECMO population. However, the American Academy of Ophthalmology recommends ROP screening for preterm infants with a birth weight of ≤ 1500 g or gestational age of ≤ 30 weeks to be conducted at 4–6 weeks post birth or at a corrected gestational age of 32 weeks [61].

### Cognitive, language, and intellectual development

ECMO has been increasingly recognized for its impact on cognitive, language, and intellectual development, particularly in children diagnosed with CDH, congenital heart disease (CHD), and those classified as critically ill newborns^[[8, 62–65]]^. There have been numerous investigations into cognitive outcomes in CDH survivors who required ECMO. Several studies have consistently indicated significantly lower cognitive composite scores in this population compared to those who did not need ECMO intervention [62, 64, 66, 67]. Danzer et al. in 2018, revealed that CDH survivors requiring ECMO scored 4.6 points lower on a cognitive composite (Bayley Scales of Infant Development, 3rd edition) [62]. In addition, Sadhwani in 2019 discovered that children who received ECMO for cardiac issues displayed significant developmental delays. Even after accounting for the primary caregiver’s education level and the number of cardiac catheterizations, a substantial difference persisted, especially in the motor domain [8]. Another study found that self-reported and parental proxy-reported PedsPCF scores (Pediatric Patient Care Function, a scale used by parents or caregivers to evaluate the care needs of children, including physical and mental health) were significantly below normal, while the total BRIEF scores (Behavior Rating Inventory of Executive Function, a comprehensive measure of a child’s executive function, as assessed by parents or teachers) were significantly above normal [65]. Therefore, these studies highlight the potential for central nervous system abnormalities and developmental delays, despite ECMO’s life-saving benefits in increasing survival rates for critically ill newborns [68].

A study examined the survival, intracranial lesions, and neurodevelopmental outcome of infants with severe CDH who underwent ECMO treatment. The results showed that 35% of the surviving infants had abnormal central nervous system findings and mild delay in cognitive development [67]. Robertson et al. found that 6 out of 40 surviving ECMO-treated infants had neurodevelopmental disabilities at two years of age compared to 1 out of 30 surviving comparison subjects. The mean mental and performance developmental indexes were similar between the ECMO and comparison groups [69]. A neonatal follow-up program by D’Agostino et al. found that the survival rate increased from 31% to 63% after the initiation of the ECMO program, with 16 out of 20 infants surviving [70]. The mean cognitive skills at one year of age were average, while the mean language skills were borderline according to the Bayley Scales of Infant Development (a standardized series of measurements used to assess the language and cognitive development of infants and toddlers), with 10 out of 13 patients having hypotonia. In conclusion, the findings of these studies suggest that ECMO treatment can lead to a high survival rate for critically ill newborns but carries a risk of central nervous system abnormalities and developmental delays [71]. Long-term neurodevelopmental outcomes of children who undergo ECMO reveal a range of outcomes, including mild cognitive and psychomotor delays, speech and language abnormalities, and cognitive impairments [72, 73]. Further research with larger multicenter studies and long-term follow-up is needed to determine the exact impact of ECMO on brain cognition [74].

Another key aspect impacted by ECMO is language development, a critical component of early childhood development. There are studies highlighting the challenges in language development among patients who underwent ECMO. For instance, research conducted by Quadir et al. on 37 patients showed that one in four infants displayed moderate to severe neurodevelopmental impairment, with language being the most impacted development domain [3]. Additionally, a study by Khachane et al., which examined infants diagnosed with CDH, found a mild delay in receptive language at one year among the cohort that underwent ECMO [4]. The effects of ECMO also extend to intellectual development and learning abilities in children. A larger study involving 3541 children with CDH by Montalva et al. found a higher incidence of neurodevelopmental impairment (NDI), including learning difficulties and neurobehavioral issues, among ECMO survivors [56]. Furthermore, a study tracked and evaluated the intelligence development of neonatal ECMO survivors up to the age of eight and its relationship with school performance. The research found that the intelligence of ECMO-surviving neonates remained stable and average at ages 2, 5, and 8, but a subset of children who received special education had below-average intelligence. The results suggest that intelligence testing alone cannot identify children with academic problems, prompting the proposal for the development of internationally standardized follow-up protocols that focus on long-term, problem-oriented neuropsychological assessment [75]. Davis argues that although the majority of ECMO-treated children appear to have "normal" intelligence in early childhood, many may still experience cognitive deficits later in life, falling behind in school during middle childhood [76]. It is important for parents of ECMO-treated children to be aware of potential developmental problems so that full assessments and interventions can be provided.

On the other hand, not all studies have reported negative outcomes associated with ECMO. The UK Collaborative ECMO trial did not observe an increase in disability at four years among ECMO recipients, and 76% of the children assessed displayed normal cognitive levels [77]. Additionally, Rais-Bahrami compared the neurodevelopmental outcomes of children treated with ECMO to those of near-miss ECMO patients, finding no significant difference in cognitive and adaptive outcomes [78]. No significant difference was found in the intelligence quotient between the reconstruction and ligation groups [79]. Long-term neuropsychological deficits were associated with the underlying disease processes in the neonatal period instead of with ECMO treatment [7]. In conclusion, while ECMO is an essential treatment that has significantly improved survival rates, it potentially impacts various neurodevelopmental outcomes. However, the evidence remains inconclusive. To fully understand the effects of ECMO on brain cognition, language development, and intellectual and learning abilities, more large-scale, multicenter studies with long-term follow-up are needed.

### Motor and behavior development

Studies have evaluated the effects of ECMO on gross motor development in infants [6, 80]. The results highlight the need for an interdisciplinary team and objective evaluation of long-term developmental problems associated with ECMO therapy [81]. Warschausky et al monitored [82] 20 infants who required ECMO and found that the mean cognitive skills were average but the mean motor skills were borderline, with 10 out of 13 patients having hypotonia. Adverse outcomes were reported in the movement assessment battery for children scores in children with CDH, sepsis, and persistent pulmonary hypertension [83]. The study by Quadir et al. found that the most significantly impacted developmental domain was gross motor function, with 62.1% of the patients affected and 7 out of 29 having severe impairment [3]. Another study by Herco et al. compared the motor function of CDH patients managed without ECMO, with one ECMO run, and with two ECMO runs [5]. The results showed that CDH neonates who underwent ECMO (single or repeat runs) were more likely to have lower motor composite scores than those who did not receive ECMO. Motor composite scores were significantly lower in repeat ECMO run neonates than in single ECMO run patients.

Most of the studies showed that children treated with ECMO had normal intelligence but had difficulties with concentration and behavior [84, 85]. A Columbia University team has shown that factors associated with lower developmental scores in children treated with ECMO include the need for ECMO, supplemental oxygen at 28 days of life, ongoing health issues, and lower socioeconomic status [86]. A follow-up study conducted in the Netherlands on five-year-old survivors of neonatal ECMO [87] showed that 17% of the children were found to have neurological deficits, with 6% having major disabilities. A study by Hibbs reviewed the outcomes of infants with bronchopulmonary dysplasia (BPD) who received ECMO therapy and found that although the survival rate was similar to or better than that of other ECMO populations, there was a high risk of severe neurobehavioral development sequelae [88]. Danzer found that neuromuscular hypotonicity is common in CDH survivors, and CDH severity was predictive of adverse neurobehavioral development outcomes [89]. These findings highlight the need for early detection and intervention of any potential developmental issues in ECMO survivors. This can include an interdisciplinary follow-up program with regular medical, neurobehavioral, and psychological assessments. Early rehabilitation and support can help improve the long-term outcomes of ECMO-treated children and ensure that they have the best chance of reaching their full potential.

### Role of nursing

#### Pretreatment support

Nurses may face the need to transfer a baby from one hospital to another for ECMO treatment. With the concept of neuroprotection in neonatal intensive care, the prevention of neuro-related complications should be considered at this time. When nurses are with the mothers and families of newborns, they can often alleviate the family’s anxiety. They can provide the infant’s family with information about the baby’s current health status and explain the current care strategies. Typically, the neonatologist or medical team responsible for the treatment would explain the potential prognosis of the infant to the parents, as well as subsequent treatment plans, assisting the parents in making decisions [90]. Considering possible long-term complications related to the nervous system, some families may decide not to initiate ECMO treatment. Teams need to respect the family’s decision while helping them understand what this may mean for their child [91].

#### Professional support

Team quality improvements may bring a positive impact on the mortality rate of critically ill patients receiving ECMO treatment through team quality improvements. The detailed methods included increasing the proportion of ECMO specialist nurses and intensivists, training nursing assistants, providing ECMO training for rehabilitative care personnel, effectively integrating multidisciplinary staff into a care team, and increasing the frequency of nursing interventions, even in patients during the infectious period of the disease [92]. It is not possible to determine the specific weight of these measures in the results, but they have implicitly reduced deaths due to complications. In addition, ECMO training and education based on high-fidelity simulation can not only improve the individual ECMO management level of nurses but also the overall level of the team [93, 94].

#### Post-treatment support

Newborns may need to stay in the hospital for a long time after ECMO treatment, which can be very difficult for families. Nurses can help families arrange accommodations, help them find family support services, and facilitate the transition to the rehabilitation phase, reducing the occurrence of neurological complications [90]. If there are long-term related complications, continuing care [91] and family follow-ups by the treatment team play a very important role in family support: parents need to know whether their child will need further surgery, whether there will be developmental delays, and whether they will need to undergo rehabilitation therapy. These are questions that parents expect a professional team to answer. Families need support to be able to accept the medical challenges they may face.

### Management strategies to protect the brain

#### Appropriate patient selection

The first step in preventing neurological complications in ECMO patients is the appropriate selection of patients for the procedure. Although some explorations have been made on ECMO application for premature infants, clear guidelines on its indications and contraindications are still lacking both domestically and abroad, with most relying on neonatal ECMO treatment guidelines [95]. Neonates with preexisting neurological conditions or those with a high risk of neurological complications may not be appropriate candidates for ECMO. Therefore, it is essential to carefully evaluate the patient’s neurological status before initiating ECMO.

#### Management of hypoxia and ischemia

The carotid artery cannulation site for venoarterial ECMO in patients 18 years old or younger was found to independently increase the odds of neurologic injury in a covariate model, which is present across all age groups [96]. The specific mechanism is not clear, but it may be related to brain tissue hypoxia and ischemia. Hypoxia and ischemia can lead to neurological complications, and it is crucial to manage these conditions promptly. ECMO provides a temporary means of support for the heart and lungs, allowing these organs to heal. However, it is important to ensure adequate oxygenation and prevent ischemia during ECMO support. This can be achieved by monitoring blood gases and maintaining appropriate levels of oxygenation.

#### Management of hemodynamics

Hemodynamic instability, common in critically ill neonates, can lead to neurological complications. O’Brien found that in infants who developed cerebral hemorrhage, higher than normal cerebral blood flow velocities were noted 2–6 days prior to the clinical recognition of bleeding [97]. The results suggest that transcranial Doppler ultrasound measuring cerebral blood flow velocity could be a noninvasive way to predict cerebral complications of ECMO and warrants further research. The study concluded that cerebral blood flow velocity measurements could be a portable and useful tool in predicting cerebral complications of ECMO [97]. Monitoring and management of hemodynamics, including blood pressure, cardiac output, and fluid balance, can prevent hypotension and ensure adequate blood flow to vital organs, including the brain.

#### Monitoring for complications

Regular monitoring for complications, including hemorrhage and seizures, is essential in preventing neurological complications. The guidelines published by the American Clinical Neurophysiology Society in 2015 recommend the use of EEG as a routine neurophysiological monitoring tool for critically ill children and neonates [98], including continuous EEG, amplitude-integrated EEG, and video EEG. A single-center retrospective study conducted continuous EEG (cEEG) monitoring for the first 24 hours in 201 pediatric ECMO cases. The results found that 12% of the cases showed severely abnormal cEEG backgrounds, which were associated with mortality. Within 3.2 hours after the start of ECMO, 16% of the cases developed status epilepticus, and a higher epileptic burden was associated with mortality. Arterial (carotid artery and aorta) ECMO types were related to focal brain damage on the same side, reflecting that abnormal hemodynamics of ECMO can cause thromboembolic damage [17]. On the other hand, near-infrared spectroscopy [99] can be used to monitor brain tissue oxygen metabolism, local oxygen saturation, and cerebral blood flow perfusion during ECMO. This aids in evaluating brain blood flow function, optimizing cerebral blood flow perfusion parameters, and predicting outcomes. However, the best method or indicator for evaluating or monitoring brain blood flow regulation has not yet been established. Some studies have shown that multimodal neurophysiological monitoring combining EEG with Doppler ultrasound or near-infrared spectroscopy [100–102] may help to accurately identify brain injury in ECMO neonates and improve their clinical outcome. Early recognition and management of these complications can prevent or minimize their impact on neurological outcomes.

#### Nutritional support [103]

For nutritional support of neonates and infants undergoing ECMO, it is recommended to perform a nutritional assessment using a method suitable for critically ill pediatric patients aiming to deliver a minimum of 1.5 gm/kg/day of protein, with a gradual increase to 3 gm/kg/day as needed, to initiate nutritional delivery within 48 hours of ECMO support or when the patient is clinically stable, to prioritize enteral nutrition and use parenteral nutrition if enteral nutrition is not possible, to closely monitor for feeding intolerance, and to follow a stepwise nutrition support algorithm to determine the feasibility of enteral nutrition versus peripheral nutrition. Adequate nutrition is crucial for neonatal growth and development, including neurological development. Providing adequate nutrition during ECMO support can prevent malnutrition and promote optimal neurological development.

#### Reconstruction and early rehabilitation

Both studies focused on the impact of carotid repair and ligation following ECMO on the prognostic outcomes in neonates. The first study revealed that most neonates maintain patency in the carotid artery after repair, with no significant difference in early neurological effects compared to the ligation group [104]. The second indicates that children who had right carotid artery repair showed a reduced incidence of abnormalities in neuroimaging and cerebral palsy, potentially improving their prognosis [79]. Prolonged immobilization can lead to muscle weakness and delays in motor development. Early rehabilitation interventions can include treatments such as touch, turning over, passive exercises, and oral training, which can help to improve their motor and cognitive development and support their overall development. In addition, growth and development assessment systems such as Bayley can assist in monitoring the growth and development of infants and toddlers, preventing some complications, and promoting the development of aspects such as motor skills and language.

#### Follow-up care

Follow-up care is critical in monitoring the long-term neurological outcomes of ECMO survivors. Regular developmental assessments can identify delays in their cognitive, motor, and language skills development and allow for early intervention to address these issues. These assessments can be performed by a pediatrician, a neurologist, or a specialist in developmental and behavioral pediatrics. Additionally, imaging assessments [105], such as MRI [106, 107] or computed tomography (CT) [108, 109] scans, can help assess the extent of the brain injury and monitor changes over time. It is important to work closely with a healthcare team to develop a personalized follow-up plan for an infant with a brain injury or at high risk of brain injury. This plan should take into account the infant’s specific needs and progress over time to ensure that they receive the best possible care and support. At the same time, the establishment of the follow-up team and the cooperation network can also be used to answer the scientific questions that have not yet been determined in neonates with ECMO.

## Data Availability

Not applicable.
